# Silver Nanoparticles Targeting the Drug Resistance Problem of *Streptococcus dysgalactiae*: Susceptibility to Antibiotics and Efflux Effect

**DOI:** 10.3390/ijms23116024

**Published:** 2022-05-27

**Authors:** Diana Garibo Ruiz, Ekaterina Nefedova, Nikolay N. Shkil, Nikolay A. Shkil, Roberto Luna Vazquez-Gomez, Alexey Pestryakov, Nina Bogdanchikova

**Affiliations:** 1 Centro de Nanociencias y Nanotecnología, Universidad Nacional Autónoma de México, Ensenada 22800, Mexico; 2 Consejo Nacional de Ciencia y Tecnología, Ciudad de México 03940, Mexico; 3 Siberian Federal Scientific Centre of Agro-BioTechnologies of the Russian Academy of Sciences, 630501 Novosibirsk, Russia; filll555@mail.ru (E.N.); nicola07@mail.ru (N.N.S.); shkil52@mail.ru (N.A.S.); 4 Escuela de Ciencias de la Salud, Universidad Autónoma de Baja California, Ensenada 22890, Mexico; rluna@uabc.edu.mx; 5 Research School of Chemistry and Applied Biomedical Sciences, Tomsk Polytechnic University, 634050 Tomsk, Russia; pestryakov2005@yandex.ru

**Keywords:** drug resistance, *Streptococcus dysgalactiae*, AgNPs, efflux effect, translational research

## Abstract

The present work is a continuation of our translational research focusing on the use of silver nanoparticles (AgNPs) to solve the global problem of antibiotic resistance. In vivo fieldwork was done with 300 breeding farm cows with serous mastitis. Ex vivo assays revealed that after cow treatment with the antibiotic drug Spectromast LC^TM^, *S.* *dysgalactiae* susceptibility to 31 antibiotics dropped by 22.9%, but after treatment with Argovit–C^TM^ AgNPs, it was raised by 13.1%. This was explained by the fact that the percentage of isolates with an efflux effect after Spectromast LC treatment resulted in an 8% increase, while Argovit-C-treatment caused a 19% decrease. The similarity of these results to our previous results on *S. aureus* isolates from mastitis cows treated with the antibiotic drug Lactobay and Argovit–C^TM^ AgNPs was shown. So, mastitis treatments with Argovit-C^TM^ AgNPs can partially return the activity of antibiotics towards *S.* *dysgalactiae* and *S. aureus*, while, in contrast, treatments with antibiotic drugs such as Spectromast LC and Lactobay enhance bacterial resistance to antibiotics. The results of this work strengthen the hope that in the future the use of AgNPs as efflux pump inhibitors will recover the activity of antibiotics, and thus will preserve the wide spectrum of antibiotics on the market.

## 1. Introduction

The ability of microorganisms to survive upon exposure to antibiotics has been accelerating in the last 60 years [1]. The negative implications of antimicrobial resistance (AMR) are directly reflected in human and animal health. In 2019, more than 1.2 million people died due to antibiotic-resistant bacterial infections [2]. Yearly economic losses in Europe were estimated to be at least EUR 1.5 billion, with more than EUR 900 million corresponding to hospital costs [3], and in the USA, yearly economic losses were 55 billion USD [4]. Due to the above, the World Health Organization has declared antibiotic resistance to be one of the priority issues in the area of health [2]. Health institutions, the scientific community, and governmental leaders at the local, national, and international levels need to address the importance of finding effective and affordable solutions to combat AMR [2]. Some alternatives to overcome AMR have been reported [5]. These alternatives include the use of (1) organic compounds (essential oils, phytochemicals, metabolites from medicinal plants, etc.), (2) biomolecules (enzymes, antibodies and bacteriocins), (3) methods such as phage therapy and (4) physicochemical procedures (i.e., non-thermal atmospheric pressure plasmas, photoinactivation, chemotherapy, etc.) [5]. Usually, organic compounds and biomolecules alone and/or combined with antibiotics show antimicrobial activity with a synergistic effect. However, these molecules are expensive and have a low storage stability [5]. Regarding phage therapy and physicochemical procedures, they are laborious, requiring sophisticated equipment and highly qualified personnel. Therefore, there is still an urgent need for the development of alternative, cost-effective, and efficient antimicrobial agents that can overcome antimicrobial resistance. Nowadays, nanomaterials have achieved notable attention as novel antimicrobial products. Silver nanoparticles (AgNPs) are the most widely studied nanoparticles in the field of medical application. So, the use of AgNPs as one of the alternatives to combat AMR is a subject of great interest. 

It should be noted that the choice of cow serous mastitis for this series of studies is due to the importance of the “One Health” approach, which consists in the achievement of optimal health for people, animals, and environment. The consequences of the spread of AMR bacteria from food animals (such as cattle, poultry, etc.) may have a negative impact on both animal health and public health. The antibiotic resistance spreads from eatable animals to humans indirectly via the food chain [6]. Mastitis is one of the most common and costly diseases in the dairy industry, causing significant economic losses of €124 ($147) per cow per year, resulting in losses of 500 million, 3 billion, and 125 billion € in Germany, the EU, and worldwide, respectively [7].

In our previous work, it was shown that the treatment of cows suffering from mastitis with Argovit-C AgNPs increased the activity of 31 antibiotics against *Staphylococcus aureus* by 11%, while antibiotic treatment decreased their activity by 25% [8]. Such effects were explained by the ability of Argovit-C AgNPs to decrease the portion of isolates with an efflux effect, while antibiotic treatment resulted in its increase [8]. The present study presents the results which are the continuation of our previous article [8], and its purpose is to figure out if the same effects will be observed for *Streptococcus* species (Gram-positive cocci belonging to the *Streptococcaceae* family). The most common *Streptococcus* species that cause bovine mastitis are *Streptococcus uberis* and *Streptococcus dysgalactiae* [9]. 

## 2. Results

### 2.1. Isolation and Identification of S. dysgalactiae

The analysis of the microbial community content in milk samples from 300 cows with mastitis is presented in Table 1. It shows that the numbers and percentages of isolates were: 150 isolates (50%) of *S. dysgalactiae*; 50 isolates (16.7%) of *S. aureus*; 30 isolates (10%) of *S. agalactiae;* 30 isolates (10%) of *S. epidermis*; 25 (8.3%) of *S. pyogenes;* and 15 isolates (5%) of *E. coli* (Table 1). The present work is focused on *S. dysgalactiae*. The results for *S. aureus* were presented in [8]. Results for the other four types of bacteria will be presented elsewhere.

Out of the 300 milk samples screened, a total of 150 of the *S. dysgalactiae* isolates (77 in the group for treatment with Spectromast LC and 73 in the group for treatment with Argovit-C) were obtained before treatment. As expected, after Spectromast LC and Argovit-C treatments, fewer isolates of *S. dysgalactiae* were obtained (72 and 52 isolates, respectively). After bacterial isolation and Gram staining, the species of streptococcus were identified. Then, the biochemical characterization for the identification of the *S. dysgalactiae* species of the Streptococcaceae family were performed (Table 1). 

### 2.2. Antibiotic Susceptibility Changes after Treatments

**Figure 1:**
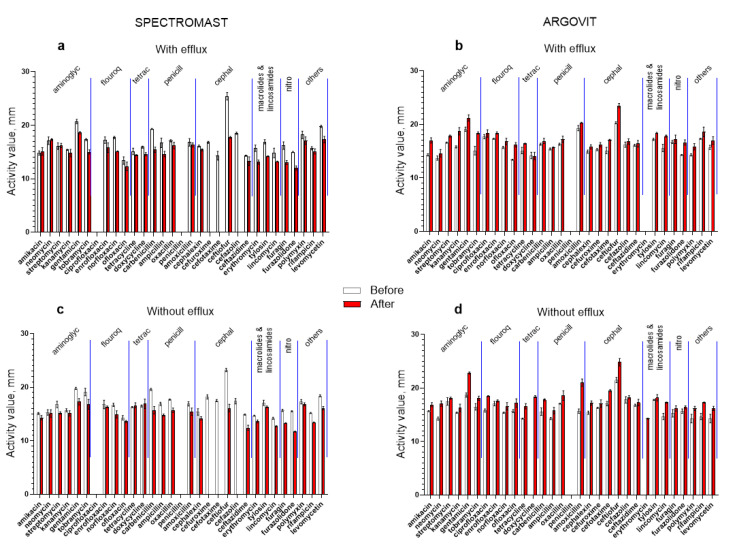
Susceptibility of *S. dysgalactiae* to 31 antibiotics before (white columns) and after (red columns) treatment with Spectromast LC (**a**,**c**) and Argovit-C (**b**,**d**). Isolates with efflux effect: (**a**) and (**b**), and without efflux effect: (**c**,**d**).

**Figure 2:**
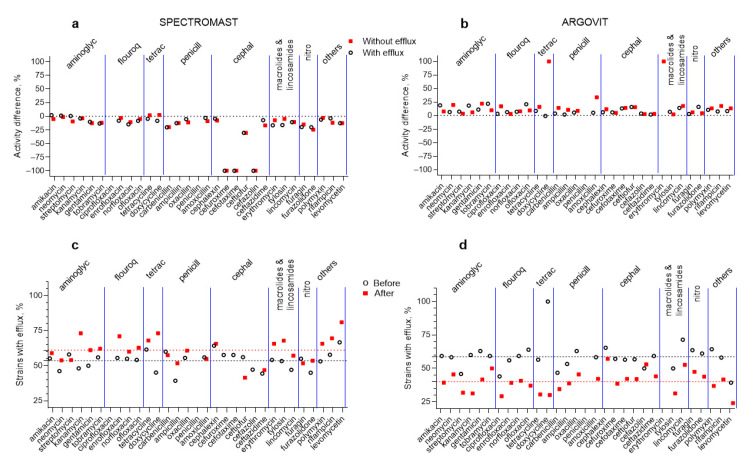
Percentage difference between the susceptibility (susceptibility value after treatment minus susceptibility value before treatment) of *S. dysgalactiae* to 31 antibiotics. Treatments: **a**—with Spectromast LC and **b**—with Argovit-C; circles—isolates with efflux effect and red squares—without efflux effect. Percentage of isolates with efflux effect for treatment with Spectromast (**c**) and Argovit-C (**d**): circles—before treatment and red squares—after treatment.

The results of the antimicrobial effect of 31 antibiotics on *S. dysgalactiae* isolates (with the efflux effect and without the efflux effect) before and after cow treatment with Spectromast LC and Argovit-C is presented in Figure 1. The percentage difference between activity before and after the treatments for each 31 antibiotics for isolates with and without the efflux effect are presented in Figure 2a,b, respectively. The results from the contribution of *S. dysgalactiae* isolates with the efflux effect before and after treatment are presented in Figure 2c,d, respectively. One of the most important obtained findings was that Spectromast LC treatment caused a notable decrease in antibiotic activity, while Argovit-C treatment led to an increase in antibiotic activity (Table 2 and Table 3, Figure 1 and Figure 2).

### 2.3. Spectromast LC

*S. dysgalactiae* isolates were not susceptible to two antibiotics (ciprofloxacin and penicillin) before and after Spectromast LC treatment. After Spectromast LC treatment, activity towards *S. dysgalactiae* isolates (with and without the efflux effect) decreased more than 22% for both isolates. After Spectromast treatment, no activity was observed for three antibiotics (cefuroxime, cefotaxime, and cefalozin) from the Cephalosporins group for isolates with and without the efflux effect (Figure 1a and Table 2). No additional changes for the rest of antibiotics after Spectromast LC treatment were observed. The antibiotic activity slightly increased (data above 0%) after Spectromast LC treatment, but only for the *S. dysgalactiae* isolates without the efflux effect, for antibiotics in the Tetracyclines group, and for isolates with the efflux effect for three antibiotics in the Aminoglycosides group (amikacin, neomycin, and streptomycin), as shown in Figure 2a and Table 2. In all other cases, the antibiotic activity decreased after Spectromast LC treatment (Figure 1a,c and Figure 2a; Table 2).

### 2.4. Argovit-C

After Argovit-C treatment, the antibiotic activity increased (for isolates with and without the efflux effect) by 13% on the average. Thus, the activity difference was positive (interval of activity difference between 0 and 100%), as seen in Figure 2b and Table 3. Activity decreased only for the isolates without the efflux effect for one antibiotic (doxycycline), as shown in Figure 2b and Table 3. *S. dysgalactiae* isolates with the efflux effect were not susceptible to penicillin and erythromycin before and after Argovit-C treatment (Figure 2b). In addition, it was observed that *S. dysgalactiae* without the efflux effect was not susceptible to penicillin.

### 2.5. Changes of the Portion of Isolates with Efflux Effect after Treatments

The changes in the percentage of *S. dysgalactiae* isolates with the efflux effect after treatment for Spectromast LC and Argovit-C are presented in Figure 2c,d, respectively. The relative changes in strains with the efflux effect increased by an average of 8% (from 54 to 60%) after Spectromast LC treatment, but in contrast, decreased by an average of 18% (from 58 to 40%) after Argovit-C treatment. Four exceptions were observed for Spectromast LC treatment (streptomycin, carbenicillin, amoxicillin, and furagin) and one for Argovit-C treatment (cefazolin). For these five antibiotics changes in the opposite direction were observed.

It is important to mention that cows with mastitis were cured after 5 days of Spectromast LC treatment and after 3 days of Argovit-C treatment.

## 3. Discussion

After Spectromast LC treatment, the susceptibility of *S.* *dysgalactiae* towards antibiotics decreased by an average of 22.9%. For three antibiotics of the cephalosporins group (cefuroxime, cefotaxime, and cefazolin), the susceptibility was lost for the isolates with and without the efflux effect; for two antibiotics (ciprofloxacin and penicillin), susceptibility was not observed, either before or after treatment.

After Argovit-C treatment, the susceptibility of *S.* *dysgalactiae* to antibiotics increased for the isolates with the efflux effect an average of only 8.9% and for the isolates without the efflux effect an average of 17.2%. Besides, the susceptibility was not lost, as in the case of Spectromast LC (for which susceptibility to three antibiotics was lost completely), but quite the opposite occurred; susceptibility to two antibiotics (doxycycline and erythromycin) appeared for the isolates without the efflux effect (Table 2 and Table 3, Figure 2). Activity remained absent for one and two antibiotics for isolates without and with the efflux effect, respectively (Table 2 and Figure 1). So, Spectromast LC treatment resulted in a 22.9% decrease in *S.* *dysgalactiae*, whereas Argovit-C treatment resulted in a 13.1% increase in the susceptibility of *S.* *dysgalactiae* isolates to 31 antibiotics (Figure 1a,b). Figure 1c,d presents the difference observed between the percentages of isolates with the efflux effect for the Spectromast LC and Argovit-C treatments. Spectromast LC treatment led to 8% growth, while Argovit-C treatment led to a 19% drop in the portion of isolates with the efflux effect. Our experimental results indicate that the treatment of mastitis with Argovit-C AgNPs can lead to a rise in *S. aureus* susceptibility to 31 antibiotics from different groups (Figure 2b). This could be due to the decrease in the portion of isolates with the efflux effect after AgNPs treatment in ex vivo experiments with cows (Figure 2d). In contrast, after treatment with Spectromast LC, *S.* *dysgalactiae* susceptibility to antibiotics decreased, and the percentage of isolates with the efflux effect increased.

**Figure 3:**
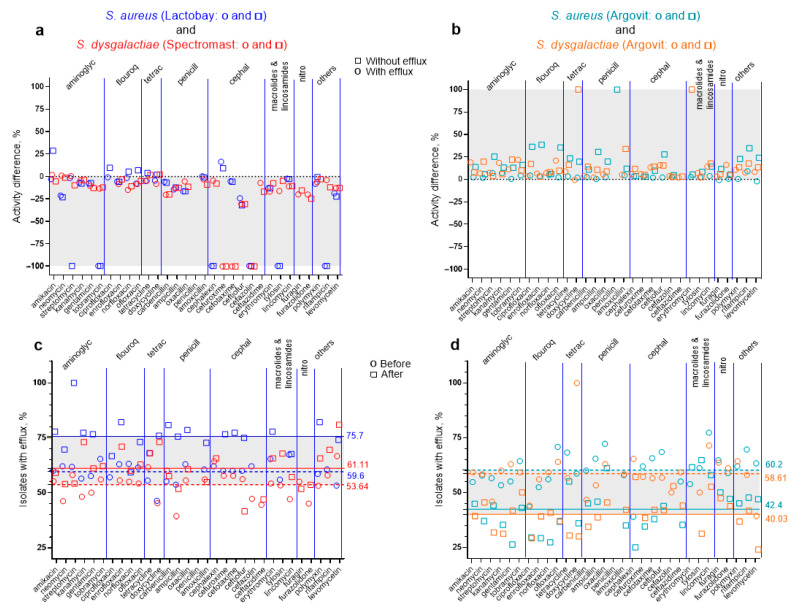
Comparison of the results for two bacteria *S. aureus* (**a**,**c**) and *S. dysgalactiae* (**b**,**d**). Circles and squares: (1) dark blue symbols correspond to Lactobay treatment; red symbols correspond to Spectromast LC treatment; light blue symbols correspond to Argovit treatment (experiments with *S. aureus*); orange symbols correspond to Argovit treatment (experiments with *S. dysgalactiae*).

These results are similar to those observed in our previous work devoted to an analogous study for *S. aureus* and the treatment of cow mastitis with Lactobay and Argovit-C [8]. Figure 3a represents the comparison of the changes in the susceptibility of *S. aureus* and *S. dysgalactiae* to antibiotics observed after treatment with Lactobay and Spectromast, respectively. Figure 3b illustrates the comparison of the changes in the susceptibility of *S. aureus* and *S. dysgalactiae* to antibiotics observed after Argovit-C treatments. The results of the comparison showed that the treatment of mastitis with antibiotic medicines Lactobay and Spectromast LC led to a 25.1 and 22.9% decrease in *S. aureus* and *S.* *dysgalactiae* susceptibility to 31 antibiotics, respectively (Figure 3a). After Argovit-C treatment, *S. aureus* and *S.* *dysgalactiae* susceptibility to antibiotics increased on average by 11.4 and 13.1%, respectively (Figure 3b). The percentage of isolates with the efflux effect for *S. aureus* increased by 17.5% after Lactobay treatment and decreased by 15% after Argovit-C treatment (Figure 3c,d), while the percentage of isolates with the efflux effect for *S.* *dysgalactiae* increased by 8% after Lactobay treatment and decreased by 19% after Argovit-C treatment (Figure 3c,d). The number of antibiotics for which the susceptibility of *S. aureus* and *S.* *dysgalactiae* remained absent or disappeared after Lactobay and Spectromast LC treatments (for isolates with and without the efflux effect) were 19 and 10 and after both Argovit-C treatments were 3, respectively (Figure 3 and [8]). All these data indicate that results for two Gram positive bacteria *S. aureus* and *S.* *dysgalactiae* have similar tendencies and close values.

It was also revealed that complete recovery from serous mastitis occurred after 6 days of Lactobay treatment and after 4 days of Argovit-C treatment [8], while in the present work complete recovery was observed after 5 and 3 days of Spectromast LC and Argovit-C treatments, respectively. Thus, the recovery with Argovit-C administration is 33 and 40% faster than recovery with the administration of Lactobay or Spectromast LC, respectively. Hence, Argovit-C treatment is more effective than treatment with Lactobay or Spectromast LC from two perspectives: reducing antibiotic resistance on the one hand and accelerating the rate of recovery on the other.

It is worth mentioning that Argovit-C has been certified for veterinary use as a therapeutic and prophylactic medication for calve gastrointestinal diseases since the year 2000. This simplifies the certification procedure of Argovit-C for mastitis treatment.

We could not find in the literature the data describing the influence of AgNPs on the susceptibility of the bacteria to antibiotics and on the bacteria efflux effect in vivo apart from our previous article dedicated to the study of *S. aureus* [8]. In the article [8], we described the data from the literature dedicated to the interactions of AgNPs and some other nanoparticle types with efflux pumps in in vitro experiments on bacterial strains of the following species: *Pseudomonas aeruginosa, Escherichia coli, Acinetobacter baumannii, Klebsiella pneumonia, Burkholderia pseudomallei*, and *Enterobacter cloacae.* The literature data showed that AgNPs in in vitro experiments can inhibit efflux pump activity and thus revive drug activity, reducing the resistance of microorganisms to these drugs [8]. However, we did not find in the literature in vitro results for the influence of AgNPs on the efflux effect for *Staphylococcus* spp. or for *Streptococcus* spp. The experiments described in the literature were undertaken in vitro, while the present work, as well as our previous one [8], are the first translational research studies, involving in vivo fieldwork with 300–400 breeding farm cows and revealing the increase in the susceptibility of two mastitis-causing bacteria to 31 antibiotics after treatments with AgNPs.

Further in vivo studies are needed to uncover if a reduction in antibiotic resistance after AgNPs treatment can be observed not only for *S. aureus* and *S.* *dysgalactiae*, but also for other bacteria and not only for mastitis, but for other diseases.

## 4. Conclusions

The present work represents the continuation of our previous translational research with the application of AgNPs as an approach to solving the worldwide drug resistance problem, in which it was revealed that cow mastitis treatment with Argovit-C AgNPs increases the susceptibility of *S. aureus* to 31 antibiotics, while treatment with the first-line commercial antibiotic drug Lactobay decreases their susceptibility. The present work revealed that the translational research approach for cow mastitis treatment with Argovit-C AgNPs and the first-line antibiotic drug Spectromast LC regarding the susceptibility of *S.* *dysgalactiae* (which is also a Gram-positive bacterium, the same as *S. aureus* studied in our previous work) led to similar results. This similarity in the results is expressed by the following data.

1. Lactobay and Spectromast LC treatments resulted in a 25.1 and 22.9% drop in susceptibility to 31 antibiotics for *S. aureus* and *S.* *dysgalactiae*, respectively.
2. Vice versa, after Argovit-C treatment, the susceptibilities of *S. aureus* and *S.* *dysgalactiae* to antibiotics increased by 11.4 and 13.1%, respectively.
3. The percentage of isolates with the efflux effect in the case of *S. aureus and S.* *dysgalactiae* dropped by 15 and 19% after Argovit-C treatments and increased by 17.5 and 8% after Lactobay and Spectromast treatments, respectively.
4. The total numbers of antibiotics for which the susceptibility of *S. aureus* and *S.* *dysgalactiae* remained absent or disappeared after treatments were 19 and 10 for Lactobay and Spectromast LC treatments, respectively, and 3 for Argovit-C treatments.
5. Mastitis recovery with Argovit-C administration occurred 33 and 40% faster than with the administration of Lactobay and Spectromast LC, respectively.
6. The changes observed in susceptibility after Argovit AgNPs and antibiotic-containing drugs (Lactobay and Spectromast LC) can be explained at least partially by the change in the percentage of isolates with the efflux effect after treatments.

The translational nature of the present study is justified because it is aiming to translate (to convert) basic study results into results that directly serve a human benefit. According to our knowledge, our translational research, performed in the previous [8] and present works, is the first in vivo fieldwork performed with 300–400 breeding farm cows that shows the possibility of decreasing the resistance of *S. aureus* and *S.* *dysgalactiae* isolates to 31 antibiotics. The results of this work strengthen the hope that, in the future, the use of AgNPs as efflux pump inhibitors will recover the activity of antibiotics, and thus will preserve the wide-spectrum of antibiotics on the market. 

## 5. Material and Methods

The experimental protocol was approved by the Ethical Committee of the Federal State Budgetary Institution of the Siberian Federal Scientific Center for Agrobiotechnologies of the Russian Academy of Sciences (decision No. 00017 from 10 February 2017).

### 5.1. Experimental Design

**Figure 4:**
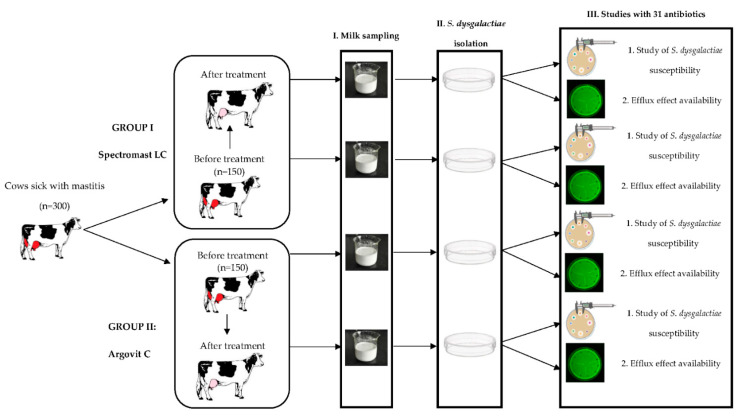
Experimental design diagram for *Str. dysgalactiae* isolates before and after treatments with Spectromast LC and Argovit-C. Stage I: milk sampling, stage II: *Str. dysgalactiae* isolation and stage III: studies of antibiotic susceptibility to 31 antibiotics and the efflux effect.

An amount of 300 dairy breeding farm cows sick with serous mastitis were included in the experiments. The mastitis diagnosis was determined by clinical symptoms with a following confirmation with the Biochemical California test [10]. Cows were divided into equal groups of 150 cows each (Figure 4). Group I involved cows treated with Spectromast LC, which is a first-line veterinary drug for mastitis treatment. The cows from group II were treated with AgNPs formulation Argovit-C^TM^. Before treatment, milk samples taken from cows from both groups were studied. These data served as a reference for both groups. Then, after both treatments, milk samples were also studied. In total, 600 milk samples were obtained: 300 samples before and 300 samples after treatments. *S. dysgalactiae* bacteria were identified and isolated from milk samples. The susceptibility of *S. dysgalactiae* isolates was studied with respect to 31 antibiotics. The scheme of the experimental design is presented in Figure 4.

### 5.2. Sample Collection

**Figure 5:**
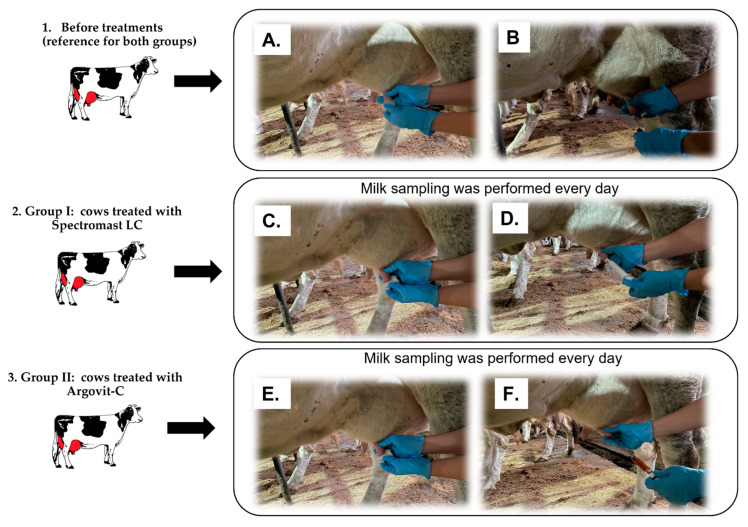
Illustration of sampling and administration of treatments to cows sick with mastitis, for each group. 1. Before treatments (reference for both groups); 2. Group I: cows treated with Spectromast LC and 3. Group II: cows treated with Argovit-C. Cleaning of teat and the teat canal surroundings (**A**,**C**,**E**), hand-stripped milk samples (**B**), intracisternal administration of Spectromast LC (**D**), and intracisternal administration of Argovit-C (**F**). Samples were always collected into sterile test tubes.

A total of six hundred samples were obtained from cows with mastitis before (*n* = 300) and after treatment with Spectromast LC (*n* = 150) and Argovit-C (*n* = 150) under conditions of livestock farms of the Novosibirsk Region during 2018–2019. For sampling before and treatments, the teats and area surrounding the teat canal were wiped with a cotton swab moistened with 70% ethyl alcohol (Figure 5A,C,E). Milk samples (10 mL each) were collected aseptically into sterile test tubes avoiding contact with the nipple by the edge of the tubes (Figure 5B) before treatment and every day. Next, they were stored at a temperature of 8–10 °C until testing. Within 3–4 h, the samples were delivered for research.

### 5.3. Treatment Formulations

For this study, treatment of cow mastitis with Spectromast LC (a first-line antibiotic-containing drug) and Argovit-C (commercial silver nanoparticles formulation applied in veterinary) were used.

Spectromast LC^TM^, Zoetis P&U LLC, Kalamazoo, MI, USA, is an antibacterial drug for intracisternal administration in the form of a suspension, containing ceftiofur hydrochloride as the main antibiotic and three excipients (microcrystalline wax, oleoyl polyoxylglycerides, and cottonseed oil). Spectromast LC treatment consisted in an intracisternal injection of 10 mL Spectromast LC twice a day in accordance with the instructions of the manufacturer. The Biochemical California test was performed during treatment every day to confirm complete recovery [10].

Argovit-C^TM^ produced by Vector-Vita Scientific and Production Center, Novosibirsk, Russia, was kindly donated by Dr. Vasily Burmistrov. Argovit-C^TM^ is a formulation used for therapeutic and prophylactic purposes against gastrointestinal infections in calves. This formulation is a stable aqueous suspension with an overall concentration of 200 mg/mL (metallic silver + stabilizers) in distilled water. The metallic silver concentration is 12 mg/mL (1.2 wt. %). AgNPs are stabilized by hydrolyzed collagen and polyvinylpyrrolidone with a total concentration of 18.8 wt. %. The remaining 80 wt. % of the weight corresponds to distilled water. A 10 mL volume of a 10-fold diluted Argovit-C intracisternal injection was administrated once a day for 4 days until complete recovery, which was justified with the Biochemical California test [10].

### 5.4. Procedure of Administration of Spectromast and Argovit

Firstly, before treatment with Spectromast LC or Argovit-C, o before sampling from cows of the reference groups the surrounding area of the teat canal and the teat end were disinfected using cotton pads moistened with 70% ethanol (Figure 5A,C,E,G). Spectromast LC (10 mL) was carefully inserted into the teat canal with a catheter according to the manufacturer’s recommendations (Figure 5D). Similarly, 10 mL of a ten-fold diluted solution of Argovit-C (with a final concentration equivalent to 0.12 mg/mL of metallic Ag) were intracisternally injected with a catheter (Figure 5F).

### 5.5. Isolation and Identification of S. dysgalactiae

*S. dysgalactiae* was isolated from the secretion of the mammary gland before and after cow treatment with the Spectromast LC or Argovit-C formulations (stage II in Figure 1). A total of 600 milk samples from the secretion of the mammary gland of the cows were considered eligible for the isolation of *S. dysgalactiae*. A selective additive Staph-Strepto Supplement (HiMedia Laboratories Pvt. Ltd., Mumbai, India) was used for the isolation of streptococci. *S. dysgalactiae* was biochemically identified considering the morphological characteristics, growth, and biochemical properties of the bacteria according to the commonly accepted methods described in *The Bergey’s Manual of Determinative Bacteriology*, 2000 [11]. It is worth mentioning that *S. dysgalactiae* was not isolated from all the samples (see Table 4 in Results section).

### 5.6. Antimicrobial Susceptibility and Efflux Testing

Overall studies of the susceptibility and efflux effect (Stage III in Figure 4) of the *S. dysgalactiae* strains isolated from cow milk samples were carried out ex vivo for 31 antibiotics associated in eight groups: 1. Aminoglycosides (amikacin, neomycin, streptomycin, kanamycin, gentamicin, tobramycin), 2. Fluoroquinolones (ciprofloxacin, enrofloxacin, norfloxacin, ofloxacin), 3. Tetracyclines (tetracycline, doxycycline), 4. Penicillins (carbenicillin, ampicillin, oxacillin, benzylpenicillin (penicillin), amoxicillin), 5. Cephalosporins (cephalexin, cefuroxime, cefotaxime, ceftiofur, cefazolin, ceftazidime), 6. Macrolides (erythromycin, tylosin) and lincosamides (lincomycin), 7. Nitrofurans (furagin, furazolidone), 8. Others (polymyxin, rifampicin, levomycetin (chloramphenicol)). Assays of *S. dysgalatiae* susceptibility to eight antibiotic groups and efflux effect tests were performed before and after cow treatment with Spectromast LC and Argovit-C.

*S. dysgalatiae* susceptibility was determined using the disc diffusion method on Mueller-Hinton agar (Bio-Rad, Hercules, CA, USA), according to The Clinical and Laboratory Standards Institute (CLSI) [12]. The disk diffusion results were interpreted based on the diameter of the inhibition zone. The efflux effect evaluation consisted in exposing *S. dysgalatiae* bacteria on Eugonic agar with ethidium bromide (1 mg/L). Then, the detection of the efflux effect was evaluated after 24 h using a transilluminator [13]. The absence of efflux activity was registered by the detection of fluorescence due to the capacity of ethidium bromide to penetrate through the cell wall of bacteria and remain inside the bacterial cell by linking to the DNA. On the contrary, when no fluorescence was registered, efflux occurred.

### 5.7. Statistical Analyses

Parametric and nonparametric analysis were performed for statistical analysis using the STATISTICA 13.3 program (StatSoft Inc., Tulsa, OK, USA). The compilation, correction, systematization of the original information, and result visualization were processed with GraphPad Software 9.0, San Diego, CA, USA.

## Figures and Tables

**Table 1:** Results from the biochemical tests of the *S. dysgalactiae* isolates from bovine mastitis.

Parameter	*S. dysgalactiae*
Gram-staining	*+*
Sodium Hippocrates	−
Phosphatase	+
ß-glucuronidase	−
α-galactosidase	−
Aesculin	−
Arginine	+
Inulin	−
Mannitol	−
Raffinose	−
Trehalose	+
Sorbitol	−
Ribose	+
Lactose	+
Growth on NaCl	6.5%
Temperature	45 °C

**Table 2:** Change in antibiotic activity towards *S. dysgalactiae* for isolates with and without the efflux effect observed after treatment with Spectromast LC.

Activity Change Category	Isolates without the Efflux Effect		Isolates with the Efflux Effect	
	Number of Antibiotics	Average Change in Activity	Number of Antibiotics	Average Change in Activity
Cumulative change				
Total activity change	29	−19.2%	29	−19.2%
Total activity change for 58 samples	−22.9%			
Changes detailed				
Activity remains absent	2	0	2	0
Activity disappeared (−100%)	3	−100%	3	−100%
Activity appeared (+100%)	0	0	0	0
Activity decreased (−Δ%)	24	−10.9%	23	−11.3%
Activity increased (+Δ%)	2	+2.1%	3	+1.3%
Activity constant (0%)	0	0	0	0

**Table 3:** Change in antibiotic activity towards *S. dysgalactiae* for isolates with and without the efflux effect observed after treatment with Argovit-C.

Activity Change Category	Isolates without the Efflux Effect		Isolates with the Efflux Effect	
	Number of Antibiotics	Average Change in Activity	Number of Antibiotics	Average Change in Activity
Cumulative change				
Total activity change	30	+17.2%	29	+8.9%
Total activity change for 59 samples	+13.1%			
Changes detailed				
Activity remained absent	1	0	2	0
Activity disappeared (−100%)	0	0	0	0
Activity appeared (+100%)	2	+100%	0	0
Activity decreased (−δ%)	0	0	1	−0.7%
Activity increased (+Δ%)	28	+11.3%	28	+9.3%
Activity constant (0%)	0	0	0	0

**Table 4:** The microbial community in milk samples from 300 cows with serous mastitis.

Microorganisms	Number of Isolates in 300 Samples	%
*S. aureus*	50	16.7
*S. epidermidis*	30	10
*S. dysgalactiae*	150	50
*S. agalactiae*	30	10
*S. pyogenes*	25	8.3
*E. coli*	15	5.0

## Data Availability

Available by request to the corresponding author.

## References

[1] Levy S.B. (1992). The Antibiotic Paradox: How Miracle Drugs Are Destroying the Miracle.

[2] Cooper B., Murray C.J., Ikuta K.S., Sharara F., Swetschinski L., Aguilar G.R., Gray A., Naghavi M., Lopez A.D., Zheng P. (2022). Global burden of bacterial antimicrobial resistance in 2019: A systematic analysis. Lancet.

[3] ECDC/EMEA (2009). The Bacterial Challenge: Time to React.

[4] Centers for Disease Control and Prevention, US Department of Health and Human Services (2013). Antibiotic Resistance Threats in the United States.

[5] Murugaiyan J., Kumar P.A., Rao G.S., Iskandar K., Hawser S., Hays J.P., Mohsen Y., Adukkadukkam S., Awuah W.A., Jose R.A.M. (2022). on behalf of the Global AMR Insights Ambassador Network. Progress in Alternative Strategies to Combat Antimicrobial Resistance: Focus on Antibiotics. Antibiotics.

[6] Marshall B.M., Levy S.B. (2011). Food animals and antimicrobials: Impacts on human health. Clin. Microbiol. Rev..

[7] Kabelitz T., Aubry E., van Vorst K., Amon T., Fulde M. (2021). The Role of Streptococcus spp. in Bovine Mastitis. Microorganisms.

[8] Nefedova E., Shkil N., Luna Vazquez-Gomez R., Garibo D., Pestryakov A., Bogdanchikova N. (2022). AgNPs Targeting the Drug Resistance Problem of Staphylococcus aureus: Susceptibility to Antibiotics and Efflux Effect. Pharmaceutics.

[9] Vélez J.R., Cameron M., Rodríguez-Lecompte J.C., Xia F., Heider L.C., Saab M., McClure J.T., Sánchez J. (2017). Whole-genome sequence analysis of antimicrobial resistance genes in Streptococcus uberis and Streptococcus dysgalactiae isolates from Canadian dairy herds. Front. Vet. Sci..

[10] Bardum D.A., Newbould F.H.S. (1961). The use of the California mastitis test for the detection of bovine mastitis. Can. Vet. J..

[11] Buchanan R.E., Gibbons N.E. (1974). Bergey’s Manual of Determinative Bacteriology.

[12] (2009). Performance Standard for Antimicrobial Disk Susceptibility Tests.

[13] Martins M., Viveiros M., Couto I. (2011). Identification of efflux pump-mediated multidrug-resistant bacteria by the ethidium bromide-agar cartwheel method. In Vivo.

